# Fast noisy long read alignment with multi-level parallelism

**DOI:** 10.1186/s12859-025-06129-w

**Published:** 2025-05-02

**Authors:** Zeyu Xia, Canqun Yang, Chenchen Peng, Yifei Guo, Yufei Guo, Tao Tang, Yingbo Cui

**Affiliations:** 1 College of Computer Science and Technology, National University of Defense Technology, 410073 Changsha, China; 2 National Supercomputer Center in Tianjin, 300457 Tianjin, China; 3 Haihe Lab of ITAI, 300457 Tianjin, China

**Keywords:** Sequence alignment, SMRT, Parallel processing, Vector-level parallelization, MPI, Heterogeneous parallelization

## Abstract

**Background:**

The advent of Single Molecule Real-Time (SMRT) sequencing has overcome many limitations of second-generation sequencing, such as limited read lengths, PCR amplification biases. However, longer reads increase data volume exponentially and high error rates make many existing alignment tools inapplicable. Additionally, a single CPU’s performance bottleneck restricts the effectiveness of alignment algorithms for SMRT sequencing.

**Results:**

To address these challenges, we introduce ParaHAT, a parallel alignment algorithm for noisy long reads. ParaHAT utilizes vector-level, thread-level, process-level, and heterogeneous parallelism. We redesign the dynamic programming matrices layouts to eliminate data dependency in the base-level alignment, enabling effective vectorization. We further enhance computational speed through heterogeneous parallel technology and implement the algorithm for multi-node computing using MPI, overcoming the computational limits of a single node.

**Conclusions:**

Performance evaluations show that ParaHAT got a 10.03x speedup in base-level alignment, with a parallel acceleration ratio and weak scalability metric of 94.61 and 98.98% on 128 nodes, respectively.

## Background

Sequence alignment is the process of aligning biological sequences in a certain manner to identify the similarities and differences between them [1]. It plays a important role in bioinformatics, such as genome assembly, variant detection and genotyping, etc [2]. The most time-consuming part of sequence alignment is the base-level alignment, which usually employs dynamic programming (DP) to find the optimal match between the two sequences [3]. For large-scale data, *seed-and-extend* algorithm is usually used to improve the speed [4]. This algorithm improves the efficiency of alignment by identifying short similar fragments, called *seeds*, within the sequences and extending these *seeds* to find longer similar regions.

While alignment algorithms have advanced over the past decades, sequence alignment remains the most time-consuming part of the entire pipeline [1]. Meanwhile, the rapid growth of alignment data has also posed higher demands on the alignment task [5, 6]. One feasible solution is to use parallel computing to accelerate alignment [7].

The widespread application of Single Molecule Real-Time (SMRT) also brings new challenges to sequence alignment [8, 9]. Compared with short reads produced by Next-Generation Sequencing (NGS), SMRT sequencing generates longer reads, often up to 10k bp. The increased read length results in a substantial increase in the computational workload for alignment [10]. Meanwhile, the high error rate in SMRT sequencing, poses further challenges to sequence alignment [11].

Since the alignment tools designed for NGS data are not suitable for noisy long reads, researchers have developed alignment tools for SMRT data. Regional Hashing-based Alignment Tool (rHAT) [12] is specifically designed for noisy long reads from SMRT. It demonstrates good robustness against high error rates and can align SMRT reads more consecutively, which could potentially facilitate downstream analysis [12].

To efficiently align noisy long reads, we proposed ParaHAT, which utilizes multi-level parallelism to accelerate alignment. ParaHAT focuses on parallel acceleration of rHAT without changing the original results. The design of ParaHAT aims at exploring the full use of various parallel technologies to maximize the performance. The contributions of our paper are as follows:

1. We analyze the computational processes of rHAT and identified the base-level alignment step as the hotspot issue. We optimize the DP formula, eliminating intra-loop dependency, and further accelerating this process with vector-level parallelism.
2. On the single node, we enhance base-level alignment with heterogeneous computing using OpenCL, allowing flexible selection of accelerators’ type and number.
3. We implement the parallel computation of ParaHAT across multiple nodes using MPI, thus breaking through the computational bottleneck of a single node. Additionally, we propose a integrated load balancing strategy and achieve effective pipeline processing across multiple nodes.
4. ParaHAT proposes a general parallel alignment framework that accelerates the process by fully utilizing vector-level, thread-level, and process-level parallelism within a single node, and extends the algorithm across multiple computing nodes to further improve alignment speed. Its base-level alignment is, on average, 2.13 times faster than minimap2. The speedup can reach up to 94.61 when using 128 nodes.

### Related work

Sequencing technology generates millions of reads [13]. To reconstruct the original genome, these reads need to be aligned to a reference genome to determine their positions in the genome [14]. Base-level alignment, based on DP formula [15], is the most basic step in sequence alignment algorithms that identifies the optimal alignment results [15]. The main drawback of DP alignment algorithms is their high time complexity [16]. For an alignment, with the reference sequence length as $L_{ref}$, the average read length as $L_{read}$, and the number of reads as $N_{read}$, the time complexity of DP alignment algorithms is $O(L_{ref} \cdot L_{read} \cdot N_{read})$ [17].

To reduce the runtime of base-level alignment, researchers have used various parallel approaches to accelerate the DP formula. Based on different levels of parallelism, existing parallel technologies are mainly classified into the following four categories.

**Fig. 1:**
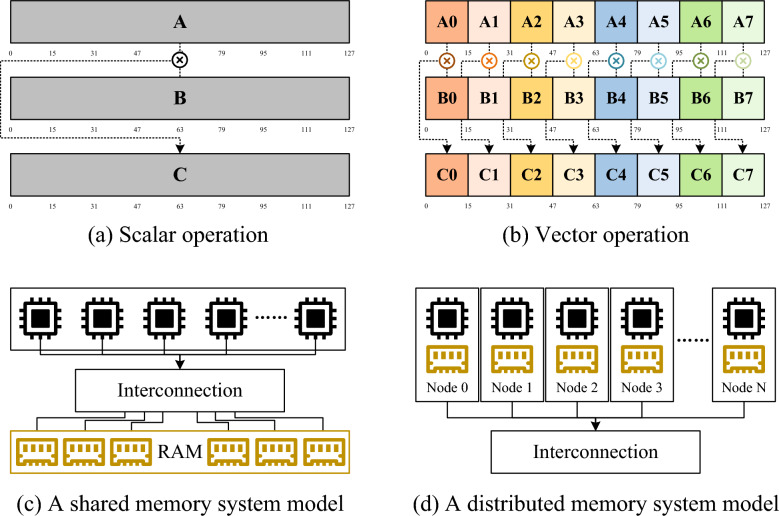
Parallel technologies. **a** Traditional serial computing using scalar operations, processing one element at a time. **b** Vector-level parallel computing using SIMD instructions, processing 8 elements at a time. **c** Schematic diagram of a shared memory system, where multiple physical cores share physical memory. **d** Schematic diagram of a distributed memory system, where computing nodes are interconnected through a network

Vector-level parallelism, also known as Single Instruction Multiple Data (SIMD), stores multiple data elements in vector registers and performs parallel operations on these data with a single instruction [18]. Figure 1a and b show the difference between traditional serial computation and SIMD. For a register of the same memory size (128 bits), serial computation can only perform operations on a single element, while SIMD with SSE vector registers can perform operations on 16 elements (each element being 8 bits) simultaneously. Intel’s SSE and AVX, ARM’s NEON, NVIDIA’s CUDA, and AMD’s OpenCL all support a specific form of the SIMD instruction set. Parasail [19] and SeqAn [20] implement vectorized parallelism of the DP formula using intra-sequence and inter-sequence parallelism, respectively, efficiently utilizing the parallel computing capabilities of the CPU. However, their vector-level parallelism is typically optimized for specific platforms to leverage SIMD characteristics and register width, leading to significant performance degradation on hardware that lacks the same vectorization support. Accel-Align [21] reduces data dependencies using low-dimensional embedding and accelerates pre-screened candidate regions using efficient SSE, achieving higher throughput in high-throughput alignment tasks. However, the embedding method may introduce errors, affecting alignment sensitivity and accuracy. WFA2-lib [22] is a high-performance alignment library based on the Wavefront Alignment (WFA) algorithm, which utilizes SIMD instructions to parallelize wavefront state updates for higher throughput [23]. However, WFA is suited for aligning highly similar sequences, and when there is significant sequence divergence, the wavefront region expands, causing a sharp increase in computation and reducing overall performance [24].

Thread-level parallelism employs a shared memory system and is one type of the Multiple Instruction, Multiple Data (MIMD) architecture. As shown in Fig. 1c, this system includes multiple physical cores that share physical memory within the computing node. In thread-level parallelism, the program is divided into multiple sub-tasks that can be executed independently, with each sub-task assigned to a thread [25]. These threads can run in parallel on a single core, multiple cores, or even different processors. Common multi-threading programming tools include POSIX Threads [25] (Pthreads) and OpenMP [26]. BWA [4] and NovoAlign [27] implement multi-threaded alignment using Pthreads to maximize the utilization of multi-core CPU. But the thread-level parallelism is limited by node performance and may encounter bottlenecks like cache contention and memory bandwidth issues as thread count increases.

Heterogeneous parallelism refers to the method of performing parallel computation in a computing environment containing different types of computing units, such as CPUs, GPUs, FPGAs, etc. It leverages the characteristics of different hardware within the system [7]. By assigning computing tasks to the most suitable hardware units to execute these tasks, heterogeneous parallelism can improve the overall computational efficiency and performance. Common heterogeneous parallel programming environments include OpenCL, CUDA, and oneAPI. OpenCL supports various processors including CPUs, GPUs, DSPs, and FPGAs. CUDA is specifically designed for NVIDIA’s GPUs. oneAPI supports Intel’s CPU, GPU, and FPGA hardware. CUSHAW [28] divides the DP formula tasks into multiple sub-tasks that can be executed in parallel, launching a large number of threads on the GPU to simultaneously compute and speed up short-read alignment. But the GPU acceleration effect is less significant for long reads or low-similarity data. GPU-BLAST [29] moves the computationally intensive local alignment of BLAST to the GPU, accelerating the overall short-read alignment process. But the data transfer and synchronization overhead between heterogeneous tasks may partially offset the benefits of GPU acceleration. Arioc [30] divides the local DP formula into multiple independent sub-tasks, performing parallel calculations on multiple candidate regions on the GPU to speed up alignment. But in some low-similarity scenarios, data dependencies and synchronization overhead may affect the acceleration performance. WFA-GPU [31] partitions the wavefront computation into parallel sub-tasks, utilizing GPU resources to accelerate sequence alignment. But for long reads with low similarity or high error rates, its performance may be affected by memory constraints and synchronization overhead, along with a decrease in accuracy.

Process-level parallelism employs a distributed memory system and is the other MIMD architecture. As shown in Fig. 1d, each computing unit has its own local memory in this system, and different units communicate with each other through a network. In process-level parallelism, data sharing and task coordination are achieved through the Message Passing Interface (MPI) [32]. MPI supports various parallel operations, including point-to-point communication and collective communication. It is suitable for parallel environments ranging from small multicore systems to large supercomputers. MSAProbs-MPI [33] uses MPI to distribute the alignment tasks across multiple computing nodes, greatly improving the efficiency of the alignment process. MpiBLAST [34] has each MPI process run the standard BLAST algorithm on its respective node. After all nodes complete local alignments, the master node collects the alignment results and merges and sorts them. Process-level parallelism is primarily affected by inter-process communication and data transfer overhead, and requires consideration of load balancing between multiple computing nodes.

rHAT is a *seed-and-extend*-based alignment tool specifically designed for noisy long reads. It indexes the reference genome using a Regional Hash Table (RHT) and uses it to find candidate positions. Afterward, it applies a sparse dynamic programming heuristic to align the reads to these positions and outputs the alignment file [12]. However, the acceleration of rHAT on modern hardware has not been fully explored. ParaHAT fills the gap by accelerating it through multi-level parallelism.

## Methods

In this section, we introduce the workflow of ParaHAT and the optimization methods used in parallel computing.

### Overview

**Fig. 2:**
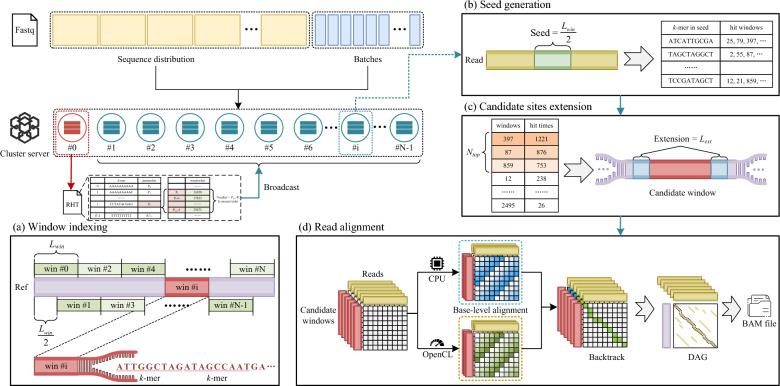
ParaHAT workflow. ParaHAT uses a dynamic load balancing strategy to distribute the sequences to be aligned across multiple nodes, which then perform the alignment and output alignment files. **a** The ParaHAT master process constructs the Regional Hash Table (RHT) of the reference sequence and broadcasts the RHT to all computing nodes. Each node uses multi-threading to generate alignment files based on the RHT and the distributed sequences. **b** ParaHAT performs seed generation, counting the window numbers in the reference genome’s RHT that contain the *k*-mers from the seeds. **c** ParaHAT performs candidate site expansion, merging and counting the hit window numbers and selecting the $N_{top}$ windows with the most hits as candidate windows. **d** ParaHAT performs read alignment using heterogeneous parallelism for base-level alignment at candidate sites and accelerates the process with vectorized parallelism. Subsequently, it traces back through the alignment results, builds a directed acyclic graph (DAG), and outputs the alignment file

The workflow of ParaHAT is shown in Fig. 2. ParaHAT uses multiple computing nodes to launch multiple processes for alignment tasks. Within a single node, ParaHAT uses multi-threading for parallel acceleration. For the most computationally intensive part of the algorithm, base-level alignment, ParaHAT accelerates the process using heterogeneous parallel computing with CPUs and accelerators, and further optimizes it with vectorized parallelism. The detailed workflow is as follows:

First, the ParaHAT master node divides the reference sequence into overlapping windows of length $L_{win}$ (default 2048 bp), with the distance between each window being $\frac{L_{win}}{2}$. Within each window, *k*-mers are extracted with a step size of *k* (usually 11$\sim$15 bp) to construct the Regional Hash Table (RHT) (Fig. 2a). The RHT consists of a PointerList and a WindowList. The PointerList for each *k*-mer stores the address of the first window number in which this *k*-mer appears in the reference genome. The WindowList stores the window numbers corresponding to each *k*-mer encoding. The master node then broadcasts the RHT to all computing nodes. Subsequently, ParaHAT adopts a dynamic load balancing strategy to evenly distribute the sequences to be aligned across the computing nodes. Each node uses multi-threading to perform alignment tasks based on the RHT and the distributed sequences in the following three steps.

The first step is seed generation (Fig. 2b). ParaHAT extracts a sequence of length $\frac{L_{win}}{2}$ from the middle of each read as the seed. For reads shorter than $\frac{L_{win}}{2}$, the entire read is used as the seed. Then, ParaHAT retrieves the window list of *k*-mers within the seed region using the RHT and calculates the *k*-mer hit count for different windows.

The second step is candidate sites extension (Fig. 2c). ParaHAT counts the hit window numbers and selects the $N_{top}$ (default 5) windows with the most hits as candidate windows. Then, it extends the candidate windows by $L_{ext}$ (default 400 bp) both forward and backward.

The third step is read alignment (Fig. 2d). ParaHAT uses the CPU and accelerators to perform base-level alignment at the candidate sites and speeds up the process with vectorized parallelism. Subsequently, it traces back through the alignment results, building a directed acyclic graph (DAG) to find the optimal path connecting the start and end positions, and outputs the alignment results.

### Vector-level parallel alignment

The vector-level parallel optimization primarily focuses on the base-level alignment in ParaHAT.

#### Base-level alignment with affine-gap penalties

ParaHAT performs global alignment based on 2-piece affine gap penalties [35], in which every residue of the two sequences is compared and aligned to find the best match across the entire sequences.

**Fig. 3:**
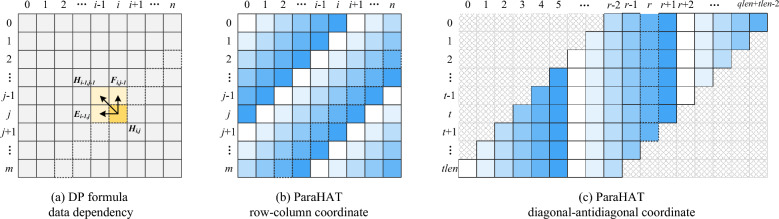
DP formula of ParaHAT. **a** Data dependency in the original DP formula. **b** The process of parallel computing in the ParaHAT algorithm’s DP formula using row and column coordinates, where cells of the same color depth are computed in parallel. **c** The process of parallel computing and indexing in the ParaHAT algorithm’s DP formula using diagonal-antidiagonal coordinates

Suppose there are two sequences to be aligned, a target sequence $S_t$ and a query sequence $S_q$. The lengths of $S_t$ and $S_q$ are $L_t$ and $L_q$, with corresponding residue indices *i* and *j*, respectively. $p_o$ and $p_o^{\prime }$ are 2 affine gap open penalty, and $p_e$ and $p_e^{\prime }$ are 2 affine gap extension penalty. A gap of length *k* costs $min\{p_o + |k| \cdot p_e, p_o^{\prime } + |k| \cdot p_e^{\prime }\}$ [35]. Function *mat*(*i*, *j*) gives the score between the two residues on $S_t$ and $S_q$. $H_{ij}$ is the score of the alignment ending at positions *i* and *j* of $S_t$ and $S_q$ prefixes. $E_{ij}$(${E^{\prime }_{ij}}$) and $F_{ij}$(${F^{\prime }_{ij}}$) are the scores with gaps ending in $S_t$ and $S_q$, respectively. The DP formula is shown in Eq. (1) [3].

1 $$\begin{aligned} \left\{ \begin{aligned} H_{i j}&=\max \left\{ H_{i-1, j-1}+mat(i, j), E_{i j}, F_{i j}\right\} \\ E_{i j}&=\max \left\{ H_{i-1, j}-p_o, E_{i-1, j}\right\} -p_e \\ F_{i j}&=\max \left\{ H_{i, j-1}-p_o, F_{i, j-1}\right\} -p_e \\ E^{\prime }_{i j}&=\max \left\{ H_{i-1, j}-p_o^{\prime }, {E^{\prime }_{i-1, j}}\right\} -p_e^{\prime } \\ F^{\prime }_{i j}&=\max \left\{ H_{i, j-1}-p_o^{\prime }, {F^{\prime }_{i, j-1}}\right\} -p_e^{\prime } \\ \end{aligned} \right. \end{aligned}$$

Computing the DP formula is a very time-consuming process. The computational complexity of the traditional serial computation method is $O(L_t \cdot L_q)$.

#### SSE vectorization

The main issue faced during the vectorized parallel computation of the DP formula is data dependency. From Formula 1 and Fig. 3a, it can be seen that the calculation of $H_{ij}$ (yellow in Fig. 3a) values depends on the values to the left cells ($E_{i-1,j}$ and $H_{i-1,j}$), above cells ($F_{i,j-1}$ and $H_{i,j-1}$), and the upper left cell ($H_{i-1, j-1}$) [36]. To address this, Wozniak [37] proposed an anti-diagonal layout, which indicates that cells along the anti-diagonal direction (dashed lines in Fig. 3a) are independent of each other, allowing for parallel computation.

The anti-diagonal layout theoretically demonstrates the feasibility of parallelizing the DP formula. However, in SSE programming, it is necessary to address the irregular data access patterns along the anti-diagonal in the DP formula. In brief, SSE vectorization requires data to be contiguous and aligned so that a block of data can be loaded into the register at once for processing [38]. But the anti-diagonal layout results in data being stored non-sequentially in memory, instead spanning multiple memory locations. Loading these scattered data elements into SSE registers from memory becomes very complex and inefficient [39]. To overcome this, ParaHAT defines $r = i + j$ and $t = i$, thereby transforming the row-column coordinate of the original DP formula into diagonal-antidiagonal coordinate. Figure 3b and c illustrate the process of coordinate transformation. Cells with the same color depth in the figure are calculated simultaneously. For cells on the same anti-diagonal (dashed lines in Fig. 3b), their original coordinates are positioned under the same vertical coordinate (dashed lines in Fig. 3c) after the coordinate transformation. This coordinate transformation ensures that data along the same diagonal is stored contiguously in memory, meeting the requirements of SSE vectorization, allowing multiple data points to be loaded simultaneously for parallel computation.

### Heterogeneous parallel alignment

Heterogeneous computing refers to the use of different types of computing units to perform computing [7]. It typically combines different hardware architectures, such as CPUs and GPUs, or specialized accelerators like FPGAs and TPUs, to maximize computational performance [40].

#### CPU + accelerators framework

**Fig. 4:**
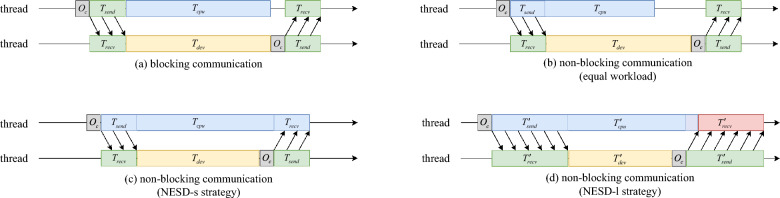
Non-equal sequence distribution optimization. **a** Blocking communication. The CPU processing time ($T_{cpu}$) and accelerators processing time ($T_{dev}$) are equal, and the program’s communication and computation processes cannot overlap. The total runtime is the sum of the communication overhead ($O_c$), communication time($T_{send} \  \&  \ T_{recv}$), and CPU processing time, i.e., $O_c + T_{send} + T_{cpu} + O_c + T_{recv}$. **b** Non-blocking communication. The CPU and accelerators processing time are equal, and the program’s communication and computation processes can overlap. The CPU need to wait for the accelerators to finish after its processing. The total runtime is the sum of the communication overhead, communication time, and accelerators processing time, i.e., $O_c + T_{send} + T_{dev} + O_c + T_{recv}$. **c** Non-blocking communication with non-equal sequence distribution strategy (NESD-s). The accelerators process shorter sequences, while the CPU processes longer sequences. The CPU processing time is equal to the sum of the communication overhead, communication time, and accelerators processing time, i.e., $T_{cpu} = T_{send} + T_{dev} + O_c + T_{recv}$. **d** Non-blocking communication with non-equal sequence distribution strategy (NESD-l). The accelerators process longer sequences, while the CPU processes shorter sequences. Both communication and computation times increase, resulting in a longer total runtime for the program

Due to the variety of accelerators and the fact that different accelerators usually use different programming models. We utilize OpenCL to ensure ParaHAT’s compatibility across various accelerators.

The design of our CPU + Accelerators framework is shown in Fig. 2d. CPU, with its robust logical control and sequential processing capabilities, manages each thread responsible for the stages of window indexing, seed generation, and candidate sites extension. For the computationally intensive base-level alignment step, we adopt a collaborative processing approach between the CPU and accelerators to accelerate it. To ensure coordinated operation between computing units in the heterogeneous system, we optimize the communication and workload between the CPU and accelerators. Additionally, we optimize the OpenCL kernel function calculating the DP formula, ensuring accelerators can utilize vectorized hardware units for acceleration.

#### Non-equal sequence distribution

To implement collaborative processing between CPU and accelerators, we launch separate threads for CPU and accelerators during base-level alignment. In heterogeneous systems, the host side (CPU) and the device side (accelerators) usually do not share memory [41]. So it is necessary to transfer data from the host to the device side and retrieve results from the device side. Since there is communication overhead in data transmission, optimizing communication within the heterogeneous system is essential. Moreover, due to varying computing performance between the host and devices, achieving load balancing between them is another issue that requires optimization.

To better explain our heterogeneous parallel alignment optimization strategy, we suppose the runtime of base-level alignment on the host and device sides is $T_{cpu}$ and $T_{dev}$, and the time for sending and receiving data between the host and device sides is $T_{send}$ and $T_{recv}$. The communication startup overhead is $O_c$, the data volume for sending and receiving are $D_{send}$ and $D_{recv}$, and the system’s data transfer bandwidth is *G*.

In heterogeneous parallelism, the transmission of data between the host and device can be divided into blocking and non-blocking communications [42]. Figure 4a shows the process of blocking communication. In blocking communication, the caller is suspended (i.e., blocked) until the communication operation completes. In this case, the program total runtime is

$$\begin{aligned} \begin{aligned} T_{total}&= O_c + T_{send} + T_{cpu} + O_c + T_{recv} \\&= 2 \cdot O_c + \frac{D_{send}+D_{recv}}{G} + T_{cpu} \end{aligned} \end{aligned}$$

In non-blocking communication, as shown in Fig. 4b, the caller can continue executing subsequent tasks without waiting for the operation to complete. In this case, the the program total runtime is

$$\begin{aligned} \begin{aligned} T_{total}&= O_c + T_{send} + T_{dev} + O_c + T_{recv} \\&= 2 \cdot O_c + \frac{D_{send}+D_{recv}}{G} + T_{dev} \end{aligned} \end{aligned}$$

It can be observed from Fig. 4b that since the computation on the device side is not shortened, the host must wait for the computational results from the device to continue the following calculation steps. This leads to no change in the total runtime of the program.

To address this, we propose a non-equal sequence distribution (NESD) strategy, allowing the workload distributed between the host and the devices unequally. This strategy fully utilizes waiting times in the program to hide more communication overhead, as shown in Fig. 4c. In this case, the total runtime is

$$\begin{aligned} \begin{aligned} T_{total} = O_c + T_{cpu}&= O_c + T_{send} + T_{dev} + O_c + T_{recv} \\&= 2 \cdot O_c + \frac{D_{send}+D_{recv}}{G} + T_{dev} \end{aligned} \end{aligned}$$

In the optimization process, we propose two sequence distribution strategies based on read lengths, evaluated through algorithm validation and experimental demonstration. The NESD-s strategy assigns shorter sequences to the accelerators and longer ones to CPU, while the NESD-l strategy does the opposite. During algorithm validation, $T_{cpu}$, $T_{dev}$, $T_{send}$, and $T_{recv}$ represent the times for each part in NESD-s strategy, and $T'_{cpu}$, $T'_{acc}$, $T'_{send}$, and $T'_{recv}$ represent the times for each part in NESD-l strategy.

Suppose there are two sequences $S_{lr}$ and $S_{sr}$ with lengths $L_{lr}$ and $L_{sr}$, respectively, where the lengths satisfy $L_{lr} = n \cdot L_{sr}$. The transmission times of $S_{lr}$ and $S_{sr}$ between the host and device sides are $T_{lr}$ and $T_{sr}$, respectively. Then, for these two sequences, their sending (receiving) DP formula sizes are $L^2_{lr}$ and $L^2_{sr}$, respectively, leading to:

$$\begin{aligned} L^2_{lr} = (n \cdot L_{sr})^2 = n^2 \cdot L^2_{sr} \end{aligned}$$

With transmitting sequences $S_{lr}$ and $n \cdot S_{sr}$ of the equivalent length between the host and device sides, their transmission times $T_{lr}$ and $n \cdot T_{sr}$ are:

$$\begin{aligned} \begin{aligned} T_{lr} = \frac{S_{lr}^2}{G}&= n^2 \cdot \frac{S_{sr}^2}{G} \\ n \cdot T_{sr}&= n \cdot \frac{S_{sr}^2}{G} \end{aligned} \end{aligned}$$

Hence $T_{lr}$ = $n \cdot n \cdot T_{sr}$. This implies that, even if the total data volume for base-level alignment remains constant, the communication time for longer sequences significantly exceeds that for shorter sequences. Thus $T'_{send} > T_{send}$, and $T'_{recv} > T_{recv}$.

Meanwhile, since accelerators usually do not match the computing performance of the CPU, the processing time on the accelerators for the same data volume in NESD-l strategy will be longer, i.e., $T'_{acc} > T_{dev}$. Therefore, NESD-l strategy needs to reduce the data volume on the device side to ensure $T'_{cpu} = T'_{acc} + T'_{send} + T'_{recv}$, which may lead to an increase in the computation time on the host side, i.e., $T'_{cpu} > T_{cpu}$. Hence, adopting NESD-l strategy, as shown in Fig. 4d, increases both computation and communication time, thereby increasing the program’s overall runtime.

Overall, processing longer sequences with the CPU and shorter sequences with accelerators can achieve better parallel performance. The comparative experimental results of the two strategies are discussed in Sect. 3.3.

#### Kernel vectorization

Different computing units, such as CPUs, GPUs, and FPGAs, typically utilize specialized vectorization capabilities. OpenCL offers a range of built-in vector data types, allowing developers to perform vectorized operations directly while writing kernel functions.

The main task of the kernel function is to implement the calculation process of base-level alignment with affine-gap penalties [35]. To achieve vectorized operations in kernel functions, we adopted the anti-diagonal layout consistent with Sect. 2.2 to eliminate the data dependency. In the SSE vectorization process, logical shift operations are used to enhance computational efficiency. For instance, we use _mm_slli_si128(xt1,1) to perform a logical left shift within a 128-bit register. Nevertheless, OpenCL does not offer a direct equivalent single function to perform this specific shift operation.

**Fig. 5:**
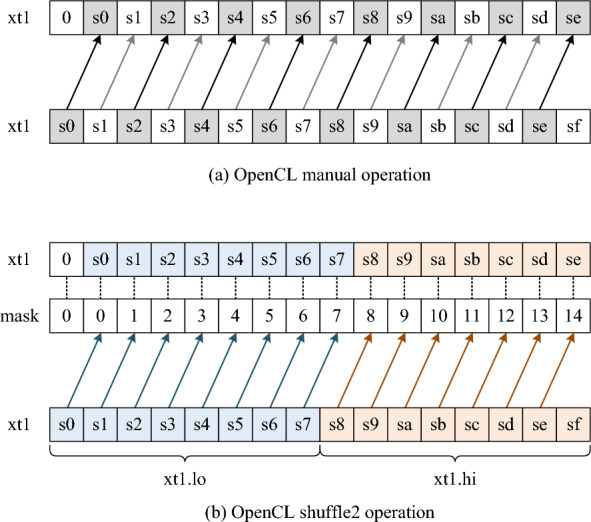
OpenCL kernel vectorization shift operation optimization. **a** OpenCL manual operation. Extract each element from the vector and assign them one by one. **b** OpenCL shuffle2 operation. Shift multiple elements simultaneously based on vector index (mask)

One solution is to simulate this shift operation with manual operations, as shown in Figrue 5a. Here, xt1.s0 to xt1.sf represent the elements of the char16 type vector xt1, covering all 16 elements (from 0 to 15). We manually specify the element for each position, and set the leftmost position to 0. This method is inefficient because it involves numerous manual operations and data copying.

To improve the efficiency of the shift operation, we utilize the shuffle2 function, as shown in Figrue 5b. It selects elements from the source vector xt1 based on the provided index vector (mask). By configuring mask with indices for a left-shifted operation and setting the first element xt1.s0 to 0, we can replicate the same shift operations as with SSE vectorization. This approach reduces the number of instructions and enhances execution efficiency compared to manual operations.

### Process-Level parallel alignment

The process-level parallelism enables the use of multiple computing nodes, significantly enhancing computational performance. The main optimization goal is to distribute the load across the nodes evenly, ensuring consistent computation times.

#### Static sequence distribution across nodes

Due to limitations in hardware performance, a single computing node may encounter performance bottlenecks. Process-level parallelism can utilize computational resources across multiple nodes, thus overcoming this limitation. Theoretically, the performance of process-level parallelism is proportional to the number of computing nodes. However, due to limitations such as inter-node communication, load imbalance, etc., the performance of process-level parallelism decreases as the number of nodes increases. To fully utilize hardware resources and enhance the overall computational performance of the system, ParaHAT optimizes the workload on each node.

ParaHAT first implements a static sequence distribution (SSD) strategy based on data volume. By pre-distributing the FASTQ files [43], the SSD strategy ensures a balanced workload on each node, thereby promoting uniform runtime across the nodes. This strategy involves two main steps: initial distribution and precise distribution. During the initial distribution, the master process calculates the data volume of the FASTQ files ($V_{fastq}$) and broadcasts it to all computing nodes via MPI_Bcast. Subsequently, each computing node calculates its own start and end positions ($Pi_s$ and $Pi_e$) based on Formula 2.

2 $$ \begin{gathered}   Pi_{s}  = \frac{{V_{{fastq}} }}{p} \cdot i \hfill \\   Pi_{e}  = \frac{{V_{{fastq}} }}{p} \cdot (i + 1) \hfill \\  \end{gathered}  $$

Where *p* is the total number of computing nodes and *i* is the index of each node. The data volume becomes equal across all nodes after the initial distribution. But the file pointers on each node might not point to the head of each sequence in the FASTQ file, potentially leading to incorrect results or even program failure. Therefore, in the precise distribution step, we relocate each pointer to the head of its respective sequence. At this point, each node obtains the correct starting position, $Pi_s$. Subsequently, adjacent nodes communicate to obtain the starting position, $Pi_s$, of the next node, thereby updating their own end position, $Pi_e$, to $Pi_s - 1$.

Considering the communication overhead in multi-node computing [44], we adopt the non-equal sequence distribution strategy, as proposed in Sect. . In this method, the master process processes more data compared to the other computing nodes.

#### Integrated load balancing strategy

The SSD strategy ensures relatively equal data volume processed by each node. But the differences in performance across computing nodes and the varying complexity of sequence computations result in significant variations in actual runtime across nodes. The node with the longest runtime becomes the primary bottleneck in algorithm performance.

So we further propose a dynamic sequence distribution (DSD) strategy to address this issue. The idea of the DSD strategy is to divide the data to be processed into smaller batches, which are then processed alternately by multiple nodes, thereby achieving better resource utilization and improving processing efficiency. An important aspect of the strategy is the selection of an appropriate batch size. A large batch size may result in significant differences in computing times between nodes, while a small batch size may require more frequent communication between nodes, increasing communication costs and thereby reducing overall execution efficiency. To address this, we integrate the DSD strategy with the SSD strategy and propose the integrated load balancing (ILB) strategy.

**Fig. 6:**
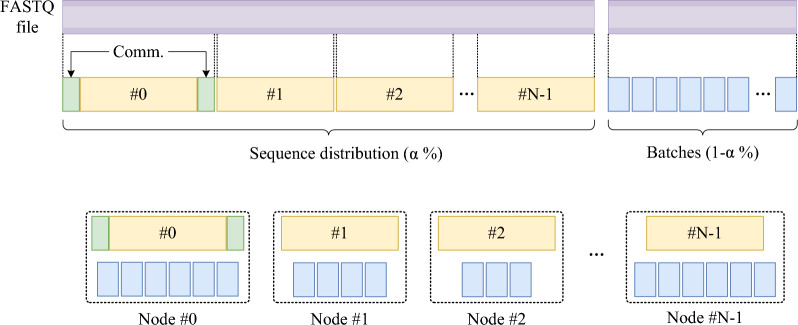
The integrated load balancing (ILB) strategy. The first $\alpha \%$ of the FASTQ file is managed with a static sequence distribution (SSD) strategy, uniformly distributing sequences across nodes and optimizing using a non-equal sequence distribution strategy. The green section in Node #0 indicates sequences processed additionally after optimization. The remaining $1 - \alpha \%$ uses a batch-based dynamic sequence distribution (DSD) strategy, dividing every *bs* sequences into a batch, with nodes processing each batch alternately

Figure 6 shows the process of the ILB strategy. The ILB strategy initially distributes the first $\alpha$ % (default 0.8) of the data evenly among the computing nodes. Then, it divides the remaining $1 - \alpha$ % of the data into batches, each consisting of *bs* (default 128) sequences. Each node alternately processes the batches in sequence after completing its computational tasks. The advantage of the ILB strategy is that, during the early stages of computation, each node does not need to spend much overhead on frequent communication, because there will not be any instances of nodes waiting for tasks. In the later stages of computation, as the amount of task data decreases, each node processes a smaller portion of data each time, thereby ensuring consistent computing time across nodes. In this strategy, the percentages ($\alpha$) of static and dynamic distribution, as well as the number of sequences (*bs*) contained in each batch, can be manually adjusted according to actual situation.

## Results

In this section, we evaluate the performance of ParaHAT. First, we analyze acceleration achieved by using the revised DP formula for SSE vectorization. Second, we discuss the acceleration effects of the CPU + Accelerators framework. Third, we measure the performance of each node on simulated and real datasets before and after implementing the dynamic load balancing strategy. Fourth, we analyze the parallel performance of ParaHAT, including the speedup, parallel efficiency, and scalability. We also compare the distribution of runtime across multiple nodes.

### Experimental setup

#### Hardware configurations

**Table 1:** Hardware configurations

Items	Local server	Cluster server
CPU	Intel i7-12900k	Intel Xeon Gold 6348
Frequency	3.9 GHz	2.6 GHz
Cores	16	28
Memory	128GB	256GB
GPU	NVIDIA RTX 3090 Ti	NVIDIA RTX A6000
MPI	openmpi-2.1.6	openmpi-2.1.6
OpenCL	3.0	3.0
OS	Ubuntu-18.04	CentOS-7.6.1810 (Core)

We conduct our experiments on a local server and a cluster server. Their hardware configurations are shown in Table 1. The local server is responsible for measuring the vector-level parallelism and heterogeneous parallel acceleration of one single node, while the cluster server is used to evaluate the performance of multi-node parallelism.

#### Datasets

We conduct benchmark tests using the same 7 real and simulated SMRT datasets as in the original rHAT paper, along with HG002 PacBio CLR, PacBio HiFi, and ONT (Oxford Nanopore Technologies) datasets. Their details are shown in Table 2. The *H. Sapiens*-real data contains raw sequence data resulting from PacBio SMRT Sequencing for CHM1TERT. The *D. melanogaster*-real data comes from a subline of the ISO1 (y; cn, bw, sp) strain of *D. melanogaster*, in collaboration with Dr. Casey Bergman at the University of Manchester and Drs. Susan Celniker and Roger Hoskins of the Berkeley Drosophila Genome Project (BDGP) at Lawrence Berkeley National Laboratory [45]. The simulated data is generated using the PBSim [46] tool. HG002 comes from the NIST’s Genome in a Bottle (GIAB) project [47]. All parameter configurations in the experiments use the default values. We randomly sample 10 million (10 M) alignment bases from each dataset to evaluate the performance of ParaHAT and other state-of-the-art (SOTA) alignment tools and libraries in the DP formula. For datasets with fewer than 10 M alignments bases, we select all available bases for evaluation. The purpose of selecting 10 M alignments is to enable both horizontal and vertical comparisons: comparing different alignment tools/libraries under the same dataset and alignment count, and evaluating the same tool/library across different datasets. Additionally, it effectively limits single-node computation time. The details of these datasets are provided in the Supplementary material Section DATA AVAILABILITY and Table S1.

**Table 2:** The real and simulated datasets information

#	Datasets	Original bases	10M alignments bases	Reference
1	*H. sapiens*-real	1810943188	46338579	hg19
2	*D. melanogaster*-real	1244028123	75042194	DM5
3	*E. coli*-sim	4938920	4938920	*E.coli* Strain 536
4	*S. cerevisiae*-sim	12153653	12153653	sacCer3
5	*D. melanogaster*-sim	129738789	75121302	DM3
6	*A. thaliana*-sim	118558112	79274551	TAIR10
7	*H. sapiens*-sim	2856372869	77637855	hg19
8	PacBio CLR	248667117073	60177420	GRCh37
9	PacBio HiFi	170093604207	117212657	GRCh37
10	ONT	194160447936	115609458	GRCh37

### Base-level alignment wall time performance evaluation

We evaluate the performance of ParaHAT with and without SSE acceleration in base-level alignment using both real and simulated data. We measure the runtime of a single thread on the DP formula for 10 M alignments across different datasets, as shown in Table 3.

**Table 3:** Comparison of timing for original DP formula, SSE acceleration, and CPU + Accelerators framework acceleration. $^{1}$

#	Datasets	Alignment	Origin	SSE	CPU+GPU
		Size	(min)	(min)	(min)
1	*E. coli*-sim	628	40.12	10.03	7.88
2	*S. cerevisiae*-sim	1569	138.32	34.09	26.30
3	*D. melanogaster*-sim	10M	640.21	163.17	125.38
4	*A. thaliana*-sim	10M	688.26	171.56	132.55
5	*H. sapiens*-sim	10M	681.53	174.07	129.78
6	*H. sapiens*-real	10M	503.05	118.59	93.30
7	*D. melanogaster*-real	10M	659.97	166.57	125.02
8	PacBio CLR	10M	839.13	202.20	126.54
9	PacBio HiFi	10M	2029.06	509.82	202.20
10	ONT	10M	2207.17	550.42	283.76

$^{1}$ All CPU executions use 1 thread

As shown in the table, ParaHAT with SSE acceleration is 3.9x to 4.2x faster than without SSE acceleration. Theoretically, a 16-way SSE vectorization can achieve up to a 16x speedup. Since the base-level alignment also includes a traceback process, and the utilization rate of vector units during computation usually cannot reach 100%, such acceleration performance is expected.

### Heterogeneous Parallel Performance

We first compare the two non-equal sequence distribution strategies in heterogeneous systems proposed in Sect. . For this purpose, we analyze the runtime of each stage in the base-level alignment, distributing sequences shorter than 1024 (NESD-s strategy) and longer than 1024 (NESD-l strategy) to the GPU on both simulated and real datasets. Then, we assess the performance of the base-level alignment before and after adopting the CPU + Accelerators framework.

#### Non-equal Sequence Distribution Strategies Comparison

**Table 4:** Comparison of time distribution for different strategies in the CPU + Accelerators framework

Strategy	Datasets	Host	Device	Comm.
		(min)	(min)	(min)
NESD-s	*E. coli*-sim	0.18	0.03	0.16
	*S. cerevisiae*-sim	0.46	0.07	0.38
	*D. melanogaster*-sim	4.85	0.75	4.28
	*A. thaliana*-sim	4.58	0.71	3.71
	*H. sapiens*-sim	19.77	4.26	15.50
	*H. sapiens*-real	5.77	1.07	5.49
	*D. melanogaster*-real	22.39	4.09	18.20
NESD-l	*E. coli*-sim	0.32	0.22	0.34
	*S. cerevisiae*-sim	0.80	0.54	0.88
	*D. melanogaster*-sim	8.51	5.76	9.54
	*A. thaliana*-sim	7.88	5.32	8.72
	*H. sapiens*-sim	34.95	23.82	61.10
	*H. sapiens*-real	10.04	6.37	12.26
	*D. melanogaster*-real	50.77	34.50	56.09

Table 4 displays the runtime of each stage under the two strategies. The runtime on the host side, device side, and communication time in NESD-l strategy increased by 1.72x to 2.27x, 5.59x to 8.44x, and 1.95x to 3.08x, respectively, compared to NESD-s strategy. Additionally, the runtime on the host side is relatively equal to the sum of the device side and communication time in NESD-s strategy, i.e., $T_{cpu} \approx T_{dev} + T_{send} + T_{recv}$. While the sum of device side and communication time is significantly greater than the host side runtime in NESD-l strategy, as shown in Fig. 4d. These results indicate that the strategy of using the CPU to process longer sequences and the GPU to process shorter sequences can better balance the workload between the host and device sides, thereby achieving a better acceleration effect.

#### CPU + accelerators framework acceleration

Subsequently, we assess the acceleration effect in base-level alignment under this sequence distribution strategy, as shown in Table 3. Compared to using only the CPU for computation, ParaHAT achieve a 1.27x to 1.34x performance improvement on the original rHAT datasets and a 1.60x to 2.52x performance improvement on the HG002 datasets. This indicates that adopting heterogeneous parallel acceleration can effectively reduce the workload on the host side, improving the overall program execution speed.

### Single node benchmark

Vector-level and heterogeneous parallelism enhance the computational performance of ParaHAT on a single node. To more accurately evaluate ParaHAT’s single-node performance, we compare it with other SOTA libraries and tools on the 10 M alignments datasets.

The alignment tools selected for the experiments include the widely used minimap2, which employs the KSW2 library for vector-level acceleration. The libraries, Parasail, WFA, and WFA-GPU, accelerate the DP formula through vector-level parallelism, WFA algorithms, and GPU-based acceleration of WFA algorithms [31], respectively. Due to differences in the alignment workflows of these tools and libraries, we focus only on comparing their runtimes during the base-level alignment. Additionally, we employ a basic Smith-Waterman-Gotoh (SWG) implementation to validate the results and calculate the recall of each method [22]. The data used in the experiments includes the original rHAT data, as well as HG002 PacBio CLR, PacBio HiFi, and ONT data. Detailed information about the data is provided in the supplementary materials.

**Table 5:** Time (*T*, in seconds) and recall (*R*, as a percentage of exact alignments) for aligners across different datasets on a single node

Dataset	Size	Metric	Minimap2	Parasail	ParaHAT	WFA	WFA-GPU
			(SSE)	(SSE)	(SSE+GPU)	(CPU)	(GPU)
*E. coli*	628	*T* (s)	69.37	110.43	46.70	3.10	2.45
		*R* (%)	100	100	100	99.99	99.68
*S. cer.*	1569	*T* (s)	153.17	269.51	105.71	6.44	5.37
		*R* (%)	100	99.93	100	100	99.29
*D. mel.* (sim)	10M	*T* (s)	795.21	1401.00	609.03	40.03	35.18
		*R* (%)	100	99.99	100	99.89	97.97
*A. tha.*	10M	*T* (s)	823.94	1453.55	541.95	45.62	39.27
		*R* (%)	100	99.99	100	100	99.07
*H. sap.* (sim)	10M	*T* (s)	795.17	1170.88	530.22	41.90	37.60
		*R* (%)	100	99.99	100	99.97	98.60
*H. sap.* (real)	10M	*T* (s)	647.86	1158.48	477.44	23.13	20.87
		*R* (%)	100	99.99	100	99.89	98.49
*D. mel.* (real)	10M	*T* (s)	767.07	1201.44	496.32	40.71	35.86
		*R* (%)	100	99.99	100	99.99	97.99
PacBio CLR	10M	*T* (s)	1604.32	3031.9	820.56	29.98	25.25
		*R* (%)	100	99.99	100	99.82	96.51
PacBio HiFi	10M	*T* (s)	2027.06	3822.86	1287.12	54.39	47.27
		*R* (%)	100	99.99	100	99.98	98.53
ONT	10M	*T* (s)	3323.89	n/a	2108.81	66.69	45.64
		*R* (%)	100	n/a	100	99.99	97.63

$^{1}$ All CPU executions use 16 threads. Executions taking more than 2 h are marked as n/a

Table 5 shows the results of ParaHAT and other SOTA alignment tools and libraries across different datasets. All experiments run on a single node and 16 threads, with tests exceeding 2 h marked as n/a. The results clearly indicate that ParaHAT outperforms minimap2 and Parasail in DP formula for both human and other ethnic genomes. This highlights ParaHAT’s adaptability, enabling fast alignment across diverse genomic datasets. Notably, ParaHAT achieves a 1.96x speedup over minimap2 for noisy PacBio CLR data, mainly due to specialized optimizations for noisy long reads and effective utilization of GPU acceleration. For high-accuracy PacBio HiFi data, ParaHAT is 1.57 times faster than minimap2, primarily due to its effective GPU acceleration strategy.

The results also show that WFA is faster than classical DP algorithms. WFA accelerates exact DP algorithms by leveraging sequence similarity, achieving a time complexity of *O*(*ns*), where *n* is the sequence length and *s* is the optimal alignment score [22]. In contrast, traditional sequence alignment algorithms typically have a complexity of $O(n^2)$. In practice, since the sequences to be aligned are often highly similar, the *s* is much smaller than *n*, making WFA significantly faster. WFA-GPU further speeds up WFA using GPUs. Its speedup on PacBio CLR, PacBio HiFi, and ONT data is similar to ParaHAT’s performance gains over minimap2, indicating that ParaHAT’s GPU acceleration performs as expected. However, WFA-GPU has a recall loss of about 2.44%, showing that WFA prioritizes speed, while classical DP algorithms focus more on accuracy and reliability.

### Effect of integrated load balancing strategy

We evaluate the impact of the ILB strategy proposed in Sect. . In this strategy, the first $\alpha$% of the data adopts the SSD strategy, while the remaining $(1 - \alpha )$% of the data is processed in batches. First, we fix the batch size *bs* to discuss the impact of $\alpha$ values on the program runtime. Second, we fix the optimal $\alpha$ value to compare the impact of different *bs* values on the program runtime. Third, we evaluate the runtime distribution of each node after using the dynamic load balancing strategy.

The experiment collects the runtime of 4 nodes, each with 2 threads, and uses bar charts with error bars to represent the runtime distribution of each node. When discussing the impact of $\alpha$ and *bs* values, we use both simulated and real datasets of *D. melanogaster*, with more experimental results available in the supplementary files.

#### Impact of $\alpha$ value

**Fig. 7:**
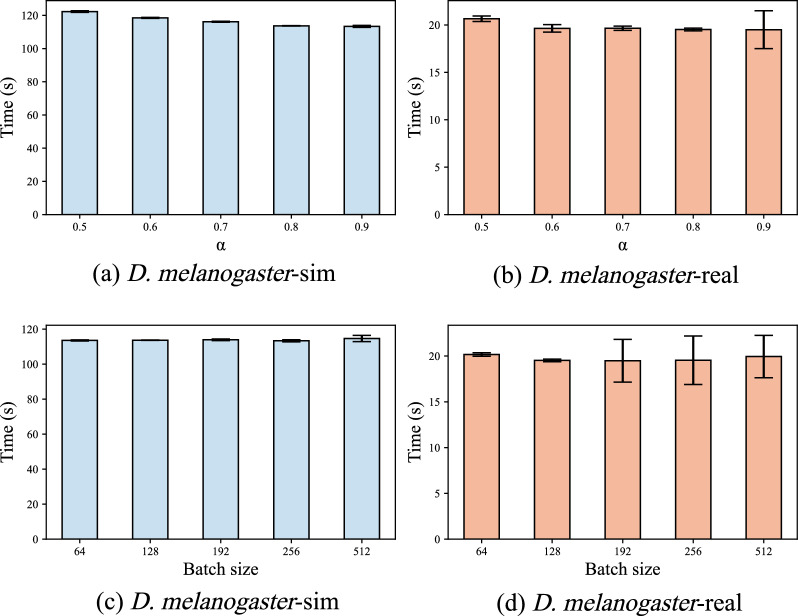
Influence of $\alpha$ and *bs* values. **a** and **b** are the impact of $\alpha$ on the program runtime on simulated and real *D. melanogaster* datasets, respectively. **c** and **d** are the impact of the *bs* value on the program runtime. The height of the bars represents the average runtime of four nodes (each with two threads), while the lower and upper ends of the error bars represent the runtime of the fastest and slowest nodes, respectively

Figure 7a and b show how different $\alpha$ values affect program runtime when $bs = 128$. As the $\alpha$ value gradually increases, the average runtime decreases and the runtime distribution across nodes becomes more balanced, achieving an optimal state at $\alpha = 0.8$. The reduction in average time mainly results from a decrease in the total number of processed batches as $\alpha$ increases, which reduces communication time across nodes. Meanwhile, frequent requests from nodes increase the workload on the master process when the $\alpha$ value is low, causing an imbalance in runtime across nodes.

As the $\alpha$ value further increases, the average runtime does not improve, and the runtime across nodes becomes imbalanced. This imbalance becomes more obvious when the dataset is small. This is because the performance of each node and the complexity of each batch computation vary, leading to different runtime for each node. And the differences become more evident with fewer batches.

#### Impact of *bs* value

Figure 7c and d show how *bs* value affects the program runtime at $\alpha = 0.8$. As the *bs* value gradually increases, the program’s average runtime and the runtime difference across nodes first decrease then increase, achieving optimal balance at $bs = 128$. This phenomenon occurs because a smaller *bs* value leads to more batches, making inter-node communication become the main hindrance to program performance. Conversely, a larger *bs* value results in fewer batches, making the differences in runtime across nodes become the main bottleneck.

#### Multi-node workload comparison

**Fig. 8:**
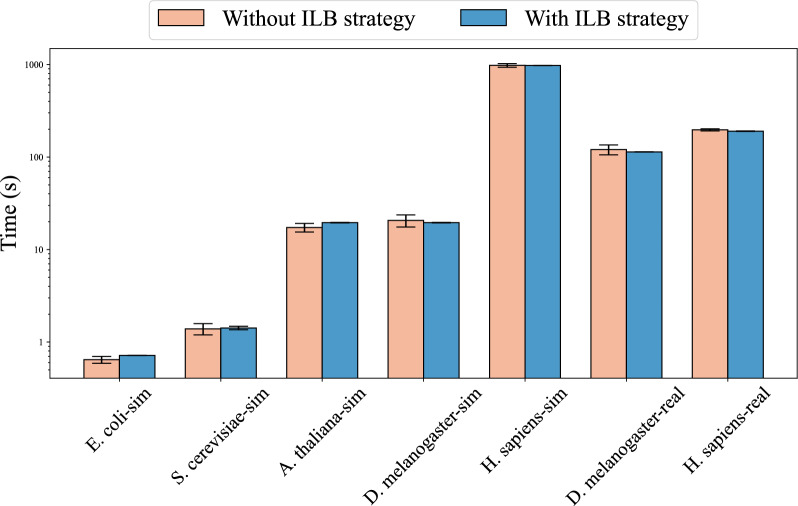
Multi-node workload comparison with and without the ILB strategy on real and simulated datasets

Figure 8 shows the differences in runtime across nodes with and without the dynamic load balancing strategy at $\alpha = 0.8$ and $bs = 128$. Compared to the SSD strategy, the ILB strategy significantly improves runtime differences across nodes. Since the performance of multiple nodes often depends on the runtime of the slowest node, the program’s performance significantly benefits from the ILB strategy. In our experiments, we note that SSD perform better with some smaller datasets due to the fact that frequent inter-node communication in the ILB strategy may reduce overall program performance. However, the data volume for actual sequence alignment is usually very large, leading us to focus more on the program’s performance with larger data volumes. The details of the other datasets influenced by the $\alpha$ and *bs* values are provided in the supplementary material Figure S1 and S2.

### Multi-node parallel performance

We evaluate the performance of ParaHAT on multiple nodes. The metrics we used are speedup, parallel efficiency, and scalability. We conduct our experiments on 1 to 128 nodes, with each node using 4 threads (maximum physical cores equal 512).

#### Speedup and efficiency

Speedup ($S_p$) is a metric for measuring the acceleration performance of parallel algorithms, defined as the ratio of the optimal execution time on a single processor ($T_s$) to the execution time on *p* processors in parallel ($T_p$), as shown in Formula 3. Ideally, the speedup is linearly related to the number of processors, i.e., $S_p = p$

3 $$\begin{aligned} S_p = \frac{T_s}{T_p} \end{aligned}$$

Parallel efficiency ($E_p$) is a metric for measuring the extent to which parallel resources are effectively utilized, defined as the ratio of speedup ($S_p$) to the number of processors (*p*), as shown in Formula 4. Ideally, parallel efficiency is 1 (or 100%), indicating that each processor is fully and effectively utilized.

4 $$\begin{aligned} E_p = \frac{S_p}{p} \end{aligned}$$

**Fig. 9:**
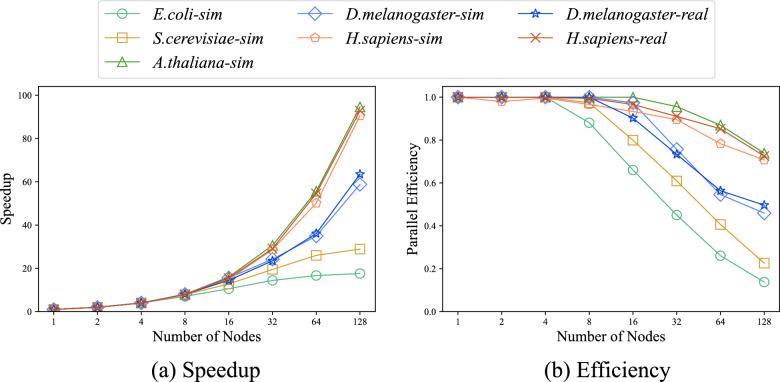
The speedup and parallel efficiency of ParaHAT. **a** Speedup ratios of ParaHAT on real and simulated datasets. The larger the dataset, the more linear the growth in speedup, peaking at 94.61x on 128 nodes. **b** Parallel efficiency of ParaHAT. The larger the dataset, the slower the reduction in parallel efficiency

Figure 9a shows the speedup of ParaHAT using both simulated and real datasets. Given that the *E. coli*-sim and *S. cerevisiae*-sim datasets are relatively small, with single-node runtimes of less than one minute, our analysis primarily focuses on the other five larger datasets. These results show that ParaHAT’s speedup exhibits a linear relationship with the number of nodes. The speedup reaches 94.61x on 128 nodes. The observed decrease in speedup is primarily due to the inter-node communication overhead. Additionally, we find that larger datasets tend to achieve higher speedup with the same number of nodes, indicating that smaller datasets have a larger proportion of time spent on communication overhead, which has a greater impact on total runtime.

To better demonstrate the parallel performance of ParaHAT, we calculate the parallel efficiency during multi-node computations, as shown in Fig. 9b. The results show that the parallel efficiency remains above 45%, peaking at 73.91% when the number of nodes reaches 128. Meanwhile, ParaHAT exhibits higher parallel efficiency at the same number of nodes with larger datasets.

#### Scalability

In the field of parallel computing, strong scalability and weak scalability are two important metrics for assessing the scalability of parallel programs.

**Fig. 10:**
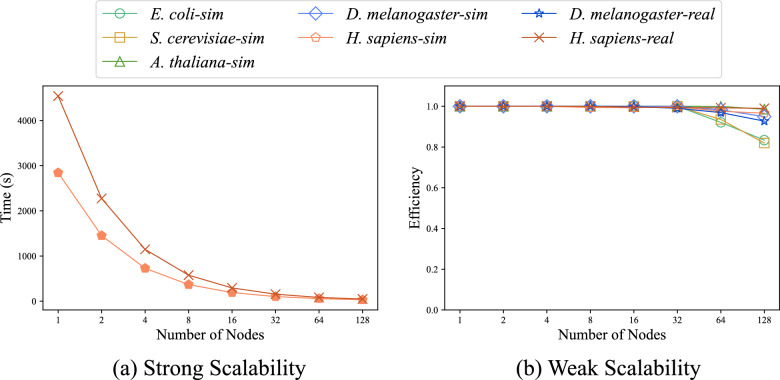
The strong and weak scalability of ParaHAT. **a** Strong scalability of ParaHAT on real and simulated *H. sapiens* datasets. The runtime of the program decreases proportionally with the number of computing nodes. **b** Weak scalability of ParaHAT on real and simulated datasets. At 128 computing nodes, only the smaller datasets *E. coli*-sim and *S. cerevisiae*-sim show a decline, with the peak weak scalability metric reaching up to 0.99

Strong scalability refers to the reduction in program runtime as the number of processors increases while keeping the total workload constant. Figure 10a and S3 show the strong scalability of ParaHAT on real and simulated *H. sapiens* and the other 5 datasets. In the experiment, we keep the dataset size constant and exponentially increased the number of nodes from 1 to 128. The results show that the program’s runtime decreases proportionally with the number of computing nodes. When using 128 nodes, the runtime decreased by 98.92% and 98.90%, respectively, such results are expected.

Weak scalability refers to how the system’s processing capability changes when the size of the problem increases linearly with the number of processors [48]. Figure 10b shows the weak scalability of ParaHAT using different datasets. In the experiments, we increase the size of the alignment files and the number of nodes in the same proportion. The results show that program runtime remains almost unchanged. When using 128 nodes, the weak scalability metric reaches 98.98%.

The above experiments indicate that ParaHAT has excellent multi-node parallel performance and is capable of efficiently handling larger-scale problems.

#### Multi-node runtime distribution

Finally, we evaluate the time distribution across different nodes by adopting a dynamic load balancing strategy. To illustrate the result more intuitively, we use violin plots to depict the time distribution of each node. The central marker, width, and length of each violin plot display the median position, frequency of occurrence, and range of distribution of the runtime, respectively.

**Fig. 11:**
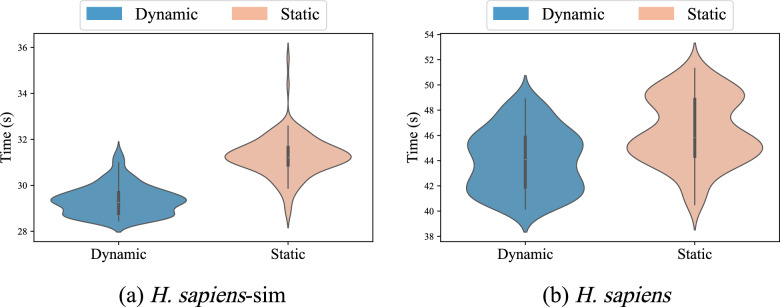
The runtime distribution of dynamic and static load balancing sequence distribution strategies. **a** and **b** illustrate the distribution of runtime across nodes using dynamic and static load balancing strategies on real and simulated *H. sapiens* datasets, respectively. The graph for the dynamic load balancing strategy appears smaller and flatter, indicating shorter runtimes and smaller variations in runtimes across nodes under this strategy

Figure 11a and b show the time distribution of 128 nodes for the real and simulated data of *H. sapiens* under dynamic and static load balancing strategies. From the results, it is evident that the coordinates at the top of the violin plots are smaller when using the dynamic load balancing strategy. This value represents the runtime of the slowest node, i.e., the actual runtime of the program. The smaller this value, the faster the multi-node program runs. Additionally, the dynamic load balancing strategy is associated with a smaller vertical span and a larger horizontal span in the plot. This indicates that the range of runtime across the nodes is more concentrated when adopting the dynamic load balancing strategy. The results of the other 5 datasets are provided in supplementary material Figure S4.

The above results show that our proposed dynamic load balancing strategy can significantly balance the workload across nodes, thereby enhancing the overall performance of the program.

## Discussion

SMRT technology has generated an unprecedented scale of biological data, thus creating an increasingly urgent demand for high-performance alignment algorithms. This paper accelerates the alignment of noisy long read sequences using parallel technologies. Specifically, we redesign the DP formula to eliminate data dependency in the base-level alignment. Subsequently, we utilize vector-level and heterogeneous parallelism to accelerate the alignment. Finally, we employ process-level parallelism to speed up the alignment on multiple nodes and propose a dynamic load balancing strategy to effectively balance the workload across nodes. Experimental results indicate that our ParaHAT exhibits excellent parallel performance, and can effectively handle large-scale alignment data.

Our experimental research shows that communication overhead in parallel computing has a significant impact on program performance. We find that high-end server CPUs remain the most effective platform for alignment tasks. Although accelerators in heterogeneous parallelism can achieve multi-way parallelism in base-level alignment, the performance of individual computing units and the communication overhead between host and devices limit the overall performance.

## Conclusions

We summarize the following recommendations for using parallel computing in sequence alignment. First, the algorithm should be highly parallelizable. Second, communication between multiple nodes, as well as between host and devices in heterogeneous parallelism, should overlap with the computing process to ensure minimal performance loss. Third, workloads should be distributed according to the computational performance of each node to prevent overall performance degradation due to differences in node runtime.

## Supplementary information

Supplementary file 1

## Data Availability

ParaHAT is available at https://github.com/nudt-bioinfo/ParaHAT. All datasets used in this paper are publicly available. The *H. Sapiens*-real data contains raw sequence data resulting from PacBio SMRT Sequencing for CHM1TERT. The *D. melanogaster*-real data comes from a subline of the ISO1 (y; cn, bw, sp) strain of *D. melanogaster*, in collaboration with Dr. Casey Bergman at the University of Manchester and Drs. Susan Celniker and Roger Hoskins of the Berkeley Drosophila Genome Project (BDGP) at Lawrence Berkeley National Laboratory [45]. HG002 comes from the NIST’s Genome in a Bottle (GIAB) project [47]. PacBio CLR dataset is available at https://ftp-trace.ncbi.nlm.nih.gov/ReferenceSamples/giab/data/AshkenazimTrio/HG002_NA24385_son/PacBio_MtSinai_NIST/PacBio_fasta/. PacBio HiFi dataset is available at https://ftp-trace.ncbi.nlm.nih.gov/ReferenceSamples/giab/data/AshkenazimTrio/HG002_NA24385_son/PacBio_CCS_15kb_20kb_chemistry2/reads/. ONT dataset is available at ftp://ftp-trace.ncbi.nlm.nih.gov/ReferenceSamples/giab/data/AshkenazimTrio/HG002_NA24385_son/Ultralong_OxfordNanopore/guppy-V3.2.4_2020-01-22/HG002_ONT-UL_GIAB_20200122.fastq.gz. Detailed information of the datasets can be found in our supplementary material section DATA AVAILABILITY.

## Abbreviations

SMRT: Single molecule real-time
DP: Dynamic programming
NGS: Next-generation sequencing
rHAT: Regional hashing-based alignment tool
bp: Base pair
SIMD: Single instruction multiple data
WFA: Wavefront alignment
MIMD: Multiple instruction, multiple data
Pthreads: POSIX threads
MPI: Message passing interface
RHT: Regional hash table
DAG: Directed acyclic graph
SSD: Static sequence distribution
DSD: Dynamic sequence distribution
ILB: Integrated load balancing
ONT: Oxford nanopore technologies
BDGP: Berkeley drosophila genome project
GIAB: Genome in a bottle
SOTA: State-of-the-art
NESD: Non-equal sequence distribution strategy
SWG: Smith-Waterman-Gotoh
