# Supplementary material for: Fast noisy long read alignment with multi-level parallelism
Source: BMC Bioinformatics. 2025 May 2;26:118. doi: 10.1186/s12859-025-06129-w (PMC12049014; doi:10.1186/s12859-025-06129-w)
Supplement: Supplementary file 1 [file 12859_2025_6129_MOESM1_ESM.pdf]

Fast Noisy Long Read Alignment with Multi-Level Parallelism

The birth of SMRT technology has resolved many limitations of second-generation sequencing, but it has also brought about an exponential increase in alignment data, as well as a higher error rate in sequencing. ParaHAT is a noise long-read alignment tool that employs multi-level parallel technology. It fully utilizes vector-level, thread-level, process-level, and heterogeneous parallelism to accelerate noise long-read alignment.

CONTENTS

1 Summary **2**

2 ParaHAT installation and usage **3**
A Requrements 3
B Installation 3
C Usage 3
D Parameters 3

3 Data availability **4**
A Availability of real datasets 4
A.1 *H. sapiens* 4
A.2 *D. melanogaster* 5
B Simulated datasets 5
C HG002 datasets 5

4 Supplementary figures **6**

5 Supplementary tables **10**

1. SUMMARY

ParaHAT integrates vector-level, thread-level, process-level, and heterogeneous parallelism to accelerate long-read sequence alignment. ParaHAT mainly focuses on parallelizing the rHAT algorithm without altering the original computational process. In the results, our parallel code achieves consistent results with the original rHAT code. The initial intention of ParaHAT is to explore the use of various parallel techniques to maximize program performance.

The parameters of ParaHAT remain consistent with the original rHAT algorithm. For detailed installation and usage instructions, refer to Section 2.

We evaluate the parallel performance of ParaHAT using datasets consistent with the original rHAT algorithm, including two real datasets and five simulated datasets. Detailed data information is provided in Section 3.

Supplementary figures provide a more detailed exposition of relevant content within the original text.

Fig. S1: The impact of α values on multi-node load balancing on 7 datasets.

Fig. S2: The impact of bs values on multi-node load balancing on 7 datasets.

Fig. S3: Strong scalability of ParaHAT on 7 datasets.

Fig. S4: The runtime distribution of dynamic and static load balancing sequence distribution strategies on 7 datasets.

Supplementary tables provide additional elaboration on the experimental information presented in the original text.

Table S1: The real and simulated datasets information.

2. PARAHAT INSTALLATION AND USAGE

Current version of ParaHAT needs to be run on Linux operating system.

A. Requrements

ParaHAT is tested to work under:

- Ubuntu 18.04
- gcc 7.5.0
- g++ 7.5.0
- openmpi 2.1.6

B. Installation

For a detailed installation process, please refer to the ParaHAT's GitHub documentation at https://github.com/xcxw127/ParaHAT

C. Usage

ParaHAT consists of two parts: indexing and alignment.

Makefile.

```
$ make clean
```

```
$ make
```

Indexing.

```
$ ./ParaHAT-indexer [-k k-merSize] <HashIndexDir> <Reference>
```

Alignment.

```
$ mpirun [-n nodeNumber] ./ParaHAT-aligner [-w windowsHits] [-m candidates] [-k
kmerSize] [-a match] [-b mismatch] [-q gapOpen] [-r gapExtension] [-t threadNumber]
<HashIndexDir> <ReadFile> <Reference>
```

D. Parameters

The basic parameters remain consistent with the original rHAT algorithm [1]. The numbers within square brackets represent default values.

./ParaHAT-indexer:

(1) `-k:` the size of the k-mers used for indexing in reference genome.[13]

./PararHAT-aligner:

(1) `-n:` the number of nodes used for multi-node running.

(2) `-w:` the max allowed number of windows hit by a k-mer.[1000]

(3) `-m:` the max number of candidates for extension.[5]

(4) `-k:` the size of the k-mer used for generating short token matches in reads.[13]

(5) `-a:` score of match for the alignments in extension phase.[1]

(6) `-b:` mismatch penalty for the alignments in extension phase.[5]

(7) `-q:` gap open penalty for the alignments in extension phase.[2]

(8) `-r:` gap extension penalty for the alignments in extension phase.[1]

(9) `-l:` the minimum length of the local matches used for SDP.[11]

(10) `-t:` the number of threads used for multi-thread running.[1]

3. DATA AVAILABILITY

A. Availability of real datasets

We evaluate the aligner using four SMRT cells from the *H. sapiens* and *D. melanogaster* datasets. Each SMRT cell consists of four reads, making a total of 12 reads.

Below are the dataset introductions, the IDs of the SMRT cells, and the download links for the read files:

A.1. H. sapiens

Dataset introduction: *H. sapiens* dataset introduction

SMRT cell 1: m130929_024849_42213_c100518541910000001823079209281311_s1_p0
Download links:

- https://s3.amazonaws.com/datasets.pacb.com/2013/Human10x/READS/2530572/0001/Analysis_Results/m130929_024849_42213_c100518541910000001823079209281311_s1_p0.1.subreads.fastq
- https://s3.amazonaws.com/datasets.pacb.com/2013/Human10x/READS/2530572/0001/Analysis_Results/m130929_024849_42213_c100518541910000001823079209281311_s1_p0.2.subreads.fastq
- https://s3.amazonaws.com/datasets.pacb.com/2013/Human10x/READS/2530572/0001/Analysis_Results/m130929_024849_42213_c100518541910000001823079209281311_s1_p0.3.subreads.fastq

SMRT cell 2: m130929_161837_42213_c100518541910000001823079209281315_s1_p0
Download links:

- https://s3.amazonaws.com/datasets.pacb.com/2013/Human10x/READS/2530572/0002/Analysis_Results/m130929_161837_42213_c100518541910000001823079209281315_s1_p0.1.subreads.fastq
- https://s3.amazonaws.com/datasets.pacb.com/2013/Human10x/READS/2530572/0002/Analysis_Results/m130929_161837_42213_c100518541910000001823079209281315_s1_p0.2.subreads.fastq
- https://s3.amazonaws.com/datasets.pacb.com/2013/Human10x/READS/2530572/0002/Analysis_Results/m130929_161837_42213_c100518541910000001823079209281315_s1_p0.3.subreads.fastq

SMRT cell 3: m130929_093545_42213_c100518541910000001823079209281313_s1_p0
Download links:

- https://s3.amazonaws.com/datasets.pacb.com/2013/Human10x/READS/2530572/0003/Analysis_Results/m130929_093545_42213_c100518541910000001823079209281313_s1_p0.1.subreads.fastq
- https://s3.amazonaws.com/datasets.pacb.com/2013/Human10x/READS/2530572/0003/Analysis_Results/m130929_093545_42213_c100518541910000001823079209281313_s1_p0.2.subreads.fastq
- https://s3.amazonaws.com/datasets.pacb.com/2013/Human10x/READS/2530572/0003/Analysis_Results/m130929_093545_42213_c100518541910000001823079209281313_s1_p0.3.subreads.fastq

SMRT cell 4: m130928_232712_42213_c100518541910000001823079209281310_s1_p0
Download links:

- https://s3.amazonaws.com/datasets.pacb.com/2013/Human10x/READS/2530572/0004/Analysis_Results/m130928_232712_42213_c100518541910000001823079209281310_s1_p0.1.subreads.fastq
- https://s3.amazonaws.com/datasets.pacb.com/2013/Human10x/READS/2530572/0004/Analysis_Results/m130928_232712_42213_c100518541910000001823079209281310_s1_p0.2.subreads.fastq

- https://s3.amazonaws.com/datasets.pacb.com/2013/Human10x/READS/2530572/0004/Analysis_Results/m130928_232712_42213_c100518541910000001823079209281310_s1_p0.3.subreads.fastq

A.2. *D. melanogaster*

Dataset introduction [2]: *D. melanogaster* dataset introduction

SMRT cell 1: m131124_190051_42175_c100583702550000001823087704281416_s1_p0
SMRT cell 2: m131124_221952_42175_c100583702550000001823087704281417_s1_p0
SMRT cell 3: m131125_013854_42175_c100583772550000001823087704281440_s1_p0
SMRT cell 4: m131125_045830_42175_c100583772550000001823087704281441_s1_p0
Download links:

- https://s3.amazonaws.com/datasets.pacb.com/2014/Drosophila/raw/Dro1_24NOV2013_398.tgz

B. Simulated datasets

The simulated data used in the experiment were generated using the PBSim tool. The P5/C3-like SMRT reads were simulated with the following command line.

```
$ pbsim --data-type CLR \
        --model_qc model_qc_clr \
        --length-mean 8000 \
        --difference-ratio 1:12:2 \
        --accuracy-mean 0.85 \
        --accuracy-min 0.8 \
        --depth 1
```

Download link for the simulated data: simulated datasets download link

C. HG002 datasets

In this study, third-generation data used include PacBio 70x (CLR), PacBio CCS 15kb_20kb chemistry2 (HiFi), and Oxford Nanopore ultralong (guppy-V3.2.4_2020-01-22) datasets. The average read length for PacBio CLR is 5,894.1 bp with a sequencing depth of 65.4349. PacBio HiFi has an average read length of 11,562.6 bp with a sequencing depth of 56.023. ONT data have an average read length of 11,043.5 bp with a sequencing depth of 56.1009.

- PacBio 70x (CLR) file can be freely downloaded from: https://ftp-trace.ncbi.nlm.nih.gov/ReferenceSamples/giab/data/AshkenazimTrio/HG002_NA24385_son/PacBio_MtSinai_NIST/PacBio_fasta/

- PacBio CCS 15kb_20kb chemistry2 (HiFi) file can be freely downloaded from: https://ftp-trace.ncbi.nlm.nih.gov/ReferenceSamples/giab/data/AshkenazimTrio/HG002_NA24385_son/PacBio_CCS_15kb_20kb_chemistry2/reads/

- Oxford Nanopore ultralong (guppy-V3.2.4_2020-01-22) can be freely downloaded from: ftp://ftp-trace.ncbi.nlm.nih.gov/ReferenceSamples/giab/data/AshkenazimTrio/HG002_NA24385_son/Ultralong_OxfordNanopore/guppy-V3.2.4_2020-01-22/HG002_ONT-UL_GIAB_20200122.fastq.gz

4. SUPPLEMENTARY FIGURES

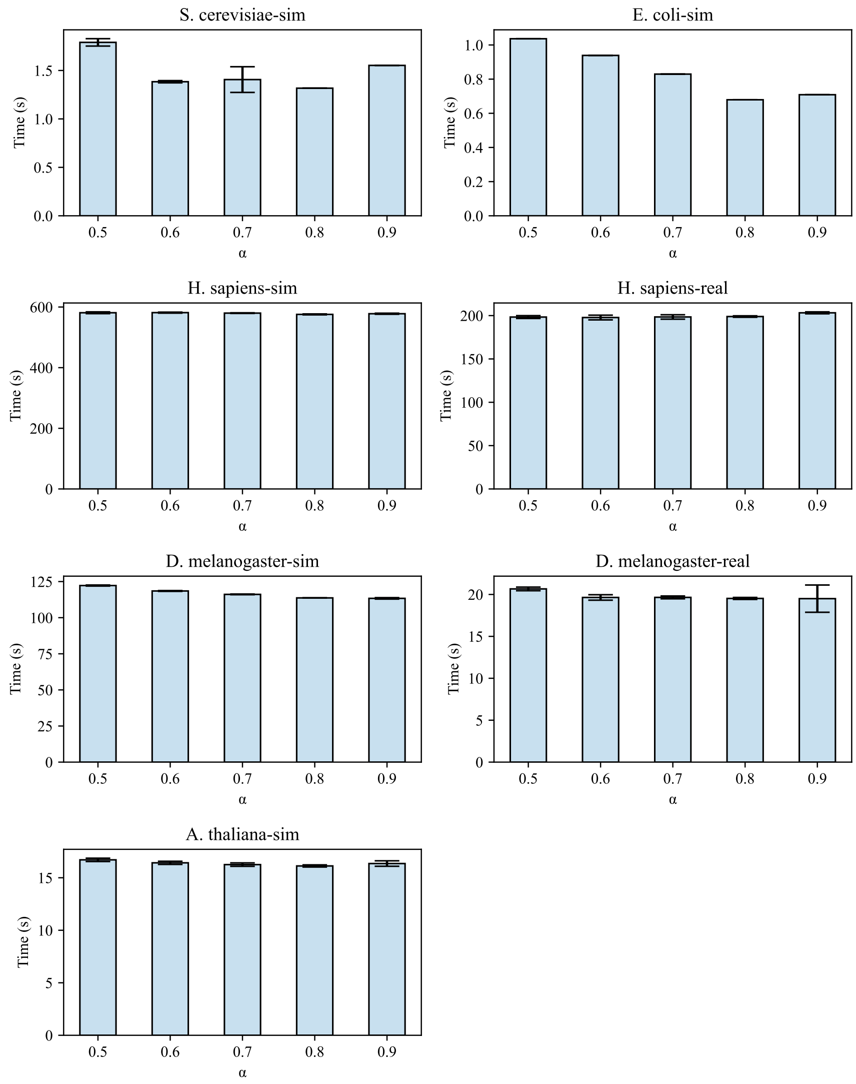

Fig. S1. The impact of α values on multi-node load balancing on 7 datasets. When the *bs* value is fixed, the difference in runtime across multiple nodes decreases initially, then increases with the value of α, reaching its optimum at $\alpha = 0.8$. When α is small, communication across nodes becomes the primary factor leading to decreased program performance. With excessively large α values, the runtime differences across nodes become more pronounced. As the runtime of a multi-node program is determined by the slowest node, this leads to a slowdown in the overall program speed.

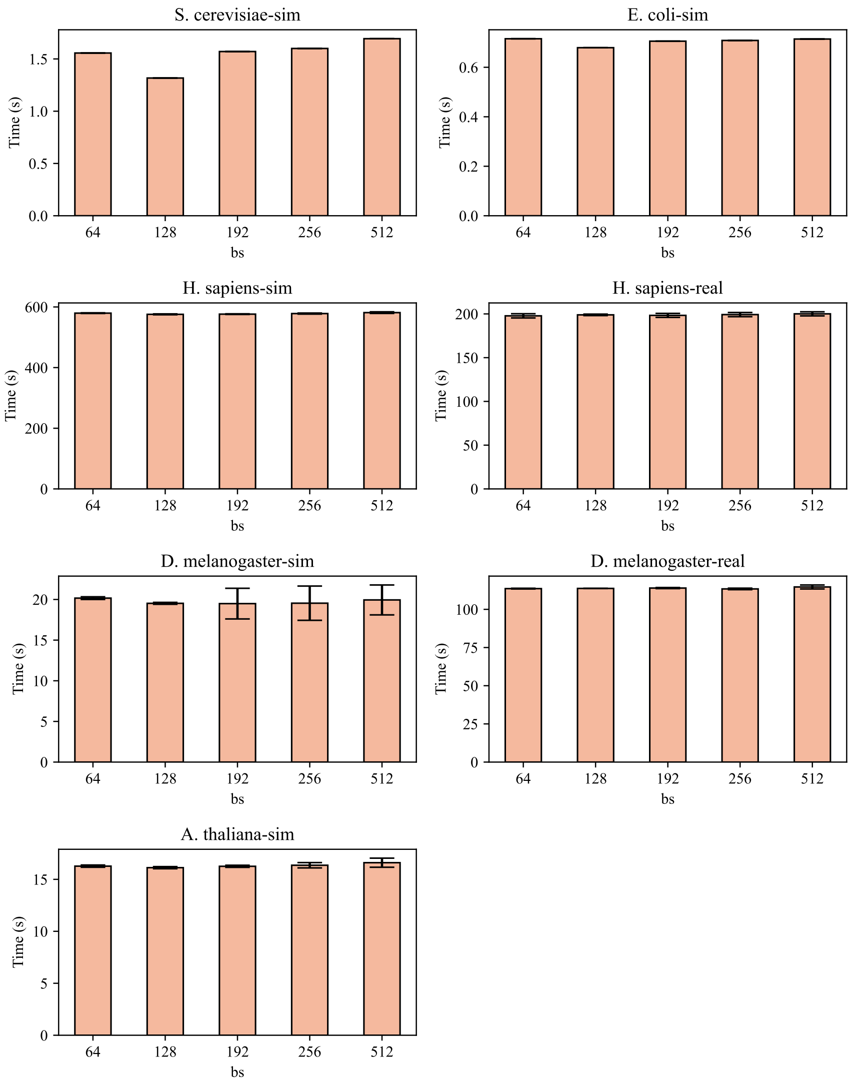

Fig. S2. The impact of *bs* values on multi-node load balancing on 7 datasets. When the α value is fixed, the difference in runtime across multiple nodes initially decreases, then increases with the *bs* value, reaching its optimum at $bs = 128$. When *bs* value is small, communication across nodes becomes the primary factor leading to decreased program performance. With excessively large *bs* values, the runtime differences across nodes become more pronounced.

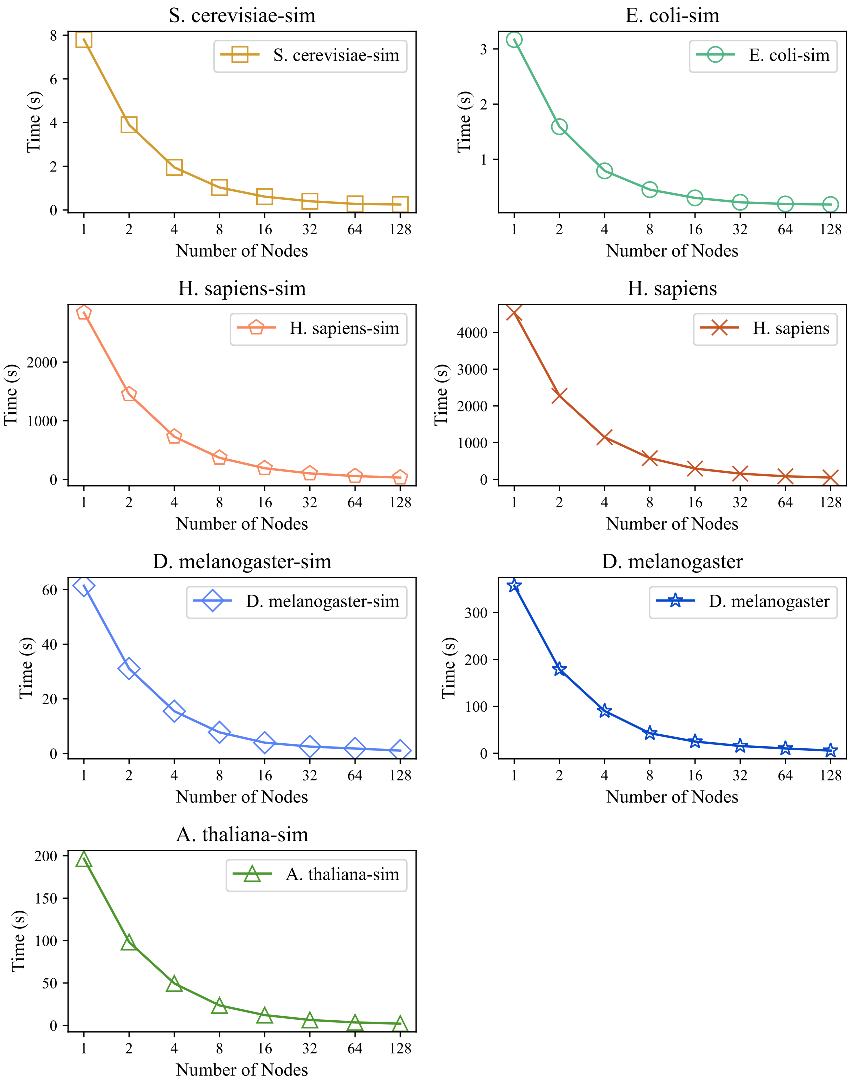

Fig. S3. Strong scalability of ParaHAT on 7 datasets. The runtime of the program decreases proportionally with the number of computing nodes, and this effect becomes more pronounced with larger datasets.

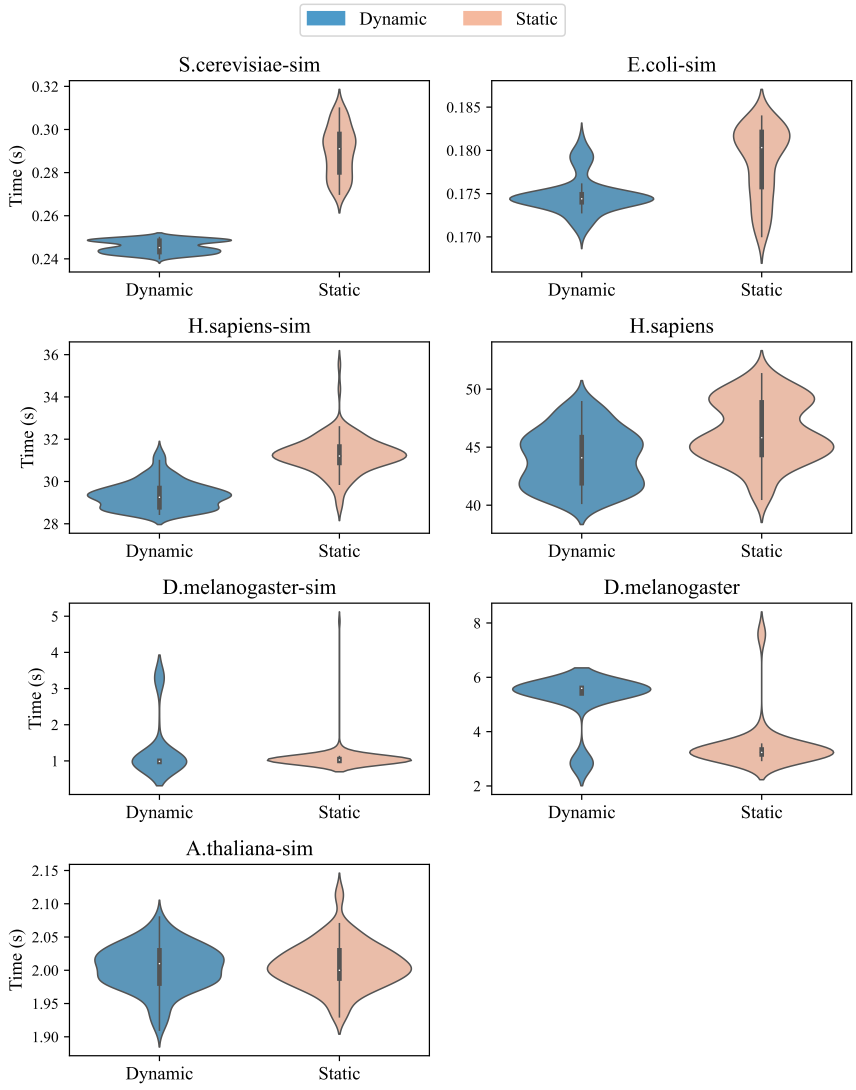

Fig. S4. The runtime distribution of dynamic and static load balancing sequence distribution strategies on 7 datasets. In the graph of the dynamic load balancing strategy, a smaller y-coordinate indicates faster runtime for the slowest node. Additionally, a shorter vertical distribution range and a wider horizontal distribution imply smaller time differences across nodes.

5. SUPPLEMENTARY TABLES

Table S1. The real and simulated datasets information

Datasets	Platform	Reads	Bases	Reference genome
H. sapiens-real	PacBio RS II P5 /C3 release	290992	1810943188	hg19
D. melanogaster-real	PacBio RS II P5 /C3 release	123234	1244028123	DM5
E. coli-sim	PBSim	623	4938920	E.coli Strain 536
S. cerevisiae-sim	PBSim	1520	12153653	sacCer3
D. melanogaster-sim	PBSim	16291	129738789	DM3
A. thaliana-sim	PBSim	14842	118558112	TAIR10
H. sapiens-sim	PBSim	357226	2856372869	hg19

REFERENCES

1. B. Liu, D. Guan, M. Teng, and Y. Wang, "rhat: fast alignment of noisy long reads with regional hashing," Bioinformatics **32**, 1625–1631 (2016).
2. K. E. Kim, P. Peluso, P. Babayan, *et al.*, "Long-read, whole-genome shotgun sequence data for five model organisms," Sci. data **1**, 1–10 (2014).
